# Surface-enhanced Raman spectroscopy-based liquid biopsy for diagnosis and classification of lupus nephritis using urine biomarkers

**DOI:** 10.3389/fimmu.2026.1808890

**Published:** 2026-05-05

**Authors:** Xue Xia, Shengyang Sun, Jiaqi Wang, Shuping Xu, Ping Li

**Affiliations:** 1 Department of Rheumatology and Immunology, China-Japan Union Hospital, Jilin University, Changchun, China; 2 School of Life Science and Technology, Changchun University of Science and Technology, Changchun, China; 3 State Key Laboratory of Supramolecular Structure and Materials, College of Chemistry, Jilin University, Changchun, China; 4 Center for Supramolecular Chemical Biology, College of Chemistry, Jilin University, Changchun, China

**Keywords:** lupus nephritis, machine learning, renal pathology, surface-enhanced Raman spectroscopy, urine

## Abstract

**Background:**

Lupus nephritis (LN) is a leading cause of mortality in patients with systemic lupus erythematosus (SLE), and the accurate classification of renal pathological subtypes is crucial for reducing mortality rates and improving long-term prognosis. Renal biopsy is the gold standard for LN diagnosis and classification; however, it is invasive, costly, and difficult to use for repeated monitoring or in all patient populations.

**Methods:**

This study established a non-invasive liquid biopsy platform based on surface-enhanced Raman spectroscopy (SERS), combined with supervised machine learning (random forest algorithm, leave-one-out cross-validation), using urine samples to achieve the diagnosis and pathological subtype classification of LN. Silver nanoparticles were used as SERS-active substrates to identify urinary biomarkers associated with LN. The study included both LN patients and those with nephrotic syndrome (NS). Machine learning algorithms were used to extract spectral features and build classification models to distinguish LN from NS. Additionally, SERS of different LN pathological subtypes were analyzed to clarify subtype-specific urinary molecular characteristics.

**Results:**

The results showed that SERS combined with machine learning can reliably and noninvasively distinguish LN from NS, achieving an LN diagnostic accuracy of 93.55%, and can stratify the main pathological subtypes of LN.

**Conclusion:**

This liquid biopsy strategy holds significant potential for non-invasive diagnosis, subtype classification, and personalized treatment decisions in LN.

## Introduction

Systemic lupus erythematosus (SLE) is a chronic autoimmune disease characterized by multisystem involvement and the presence of multiple autoantibodies (1). It often affects the kidneys and leads to lupus nephritis (LN), a severe complication and major cause of mortality in SLE patients (2). The World Health Organization first classified lupus nephritis in 1975, several significant revisions to the classification have been made since then (3). Although renal biopsy remains the gold standard for diagnosing and classifying LN, it is invasive, costly, and unsuitable for continuous monitoring. With the rise of various omics technologies, researchers have extensively studied and applied conventional biomarkers, such as autoantibodies, proteinuria, and the urinary protein-to-creatinine ratio in LN (4).

Raman spectroscopy is a powerful analytical tool that has garnered considerable attention in biomedical analysis and imaging due to its sensitivity, specificity, and multiplexing capabilities (5). It distinguishes between tumorous and non-tumorous tissues and supports the differential diagnosis of dysplasia and inflammation. This helps with early, personalized treatment recommendations (6). Surface-enhanced Raman spectroscopy (SERS) is an enhanced form that significantly amplifies the Raman signal using metallic nanostructures, such as gold or silver nanoparticles. This technique enables the detection of molecules at extremely low concentrations, even down to the single-molecule level, making it highly promising for biomedical applications (7). In recent years, SERS has been steadily used in clinical diagnostics. For example, SERS facilitates early cancer detection, pathogen identification, drug metabolism monitoring, and the detection of highly sensitive biomarkers (8). Particularly in urine analysis, SERS can provide detailed information about metabolites, proteins, and other biomolecules, offering robust support for disease diagnosis and monitoring (9). Compared to traditional biochemical analysis methods, SERS offers advantages such as rapid analysis, non-invasiveness, and high throughput, demonstrating significant potential in clinical diagnostics (10).

**Scheme 1:**
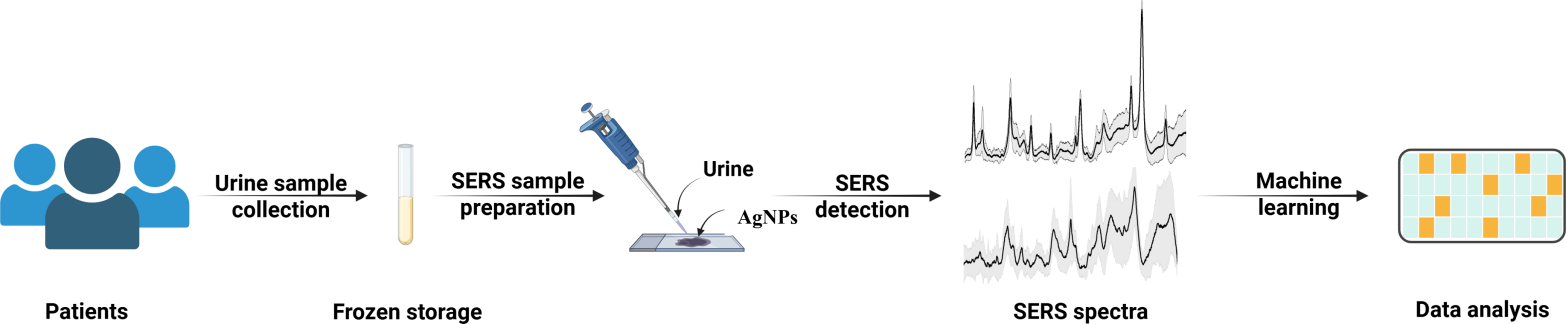
SERS technology combined with machine learning to analyze urine samples from patients with LN and NS.

Urine sampling is a non-invasive method that is easier to collect than blood or tissue samples. Since urine is reabsorbed and concentrated in the kidneys, it reflects the pathological changes in the kidneys. Several studies have reported the use of Raman spectroscopy to analyze urine and blood samples from patients with renal failure (11), renal transplantation (12), diabetic nephropathy (13), and other diseases. In this study, we used label-free SERS to compare urine samples from LN and NS patients, aiming to identify potential differences between the two diseases. We used machine learning algorithms to construct a classification model to distinguish between LN and NS. Finally, we classified the samples based on different types of kidney pathology and explored the differences in urine composition accordingly (**Scheme 1**).

## Materials and methods

### Patient cohort

This study strictly followed the ethical guidelines of the Human Research Ethics Committee of the China-Japan Union Hospital of Jilin University (2024071814). Informed consent was obtained from all participants before sample collection. The LN group consisted of 31 patients (7 males and 23 females, with an average age of 43 ± 11 years), recruited from the outpatient and inpatient rheumatology and immunology departments between July 2023 and December 2024. The control group consisted of 16 NS patients (13 males and 3 females, with an average age of 55 ± 2 years; **Table 1**). All patients repeatedly showed urine protein 4+ on urinalysis, and 24-hour urinary protein quantification revealed heavy proteinuria, with newly diagnosed cases and no prior treatment history. Serum creatinine levels in all enrolled patients were within the normal range. The urine pH and specific gravity of the enrolled patients were within normal ranges.

**Table 1:** The simple information of patients with NS and LN.

Characteristics	NS(n=16)	LN(n=31)
Age		
Mean	55	43
Standard deviation	2	11
Gender		
Male	13	7
Female	3	24

In the LN group, 18 patients underwent renal biopsy, which is the gold standard for the diagnosis and classification (**Table 2**). Histological classification was based on the “2018 Revised International Society of Nephrology/Renal Pathology Society (ISN/RPS) Classification Criteria”. All biopsy specimens were independently evaluated by at least two experienced renal pathologists at our center, and the final diagnosis was determined by consensus.

**Table 2:** Number of lupus nephritis patients by pathological class.

Pathological class	Number	Spectra no
Minimal mesangial lupus nephritis (I-LN)	2	100
Focal lupus nephritis (III-LN)	2	100
Diffuse lupus nephritis (IV-LN)	2	100
Membranous lupus nephritis (V-LN)	5	250
III+IV-LN	3	150
IV+V-LN	4	200
Total	18	900

### Collection, processing, and storage of urine samples

Morning urine was collected after an 8-hour fast from LN and NS patients. 10 mL were divided into 1.5 mL tubes, each with 500 µL urine, and stored at -80 °C for SERS analysis.

### Sample preparation for surface-enhanced Raman spectroscopy

Among various SERS-active nanomaterials, silver-based nanostructures were selected as SERS probes in this study. Compared to other metal nanoparticles, this material is inexpensive, easy to synthesize, and exhibits good biocompatibility (14). Silver nanoparticles (AgNPs) colloids, prepared by the citrate reduction method (15), were characterized by scanning electron microscopy (SEM) and dynamic light scattering (DLS). Three microliters of AgNPs were pipetted onto a clean glass slide. The slide was then placed on a thermostatic heater at 60 °C and dried for about 20 minutes. Urine samples were thawed to room temperature. Three microliters of each sample were pipetted onto the slide to cover the AgNPs.

### Surface-enhanced Raman spectroscopy measurements

SERS measurements were made using a confocal Raman system. On each experimental day, before running patient samples, we calibrated the slide and collected the SERS of a standard reference medium. We monitored the intensity and peak position of this standard to ensure the consistency of the instrument and SERS substrate performance. Urine samples were placed on the sample plane of a 633 nm laser line-scan Raman microscopy system. A 50×, 0.75 NA objective lens focused the liquid samples. The laser wavelength was 633 nm, and the power was set to 7.0 mW. The grating specification was 1800 grooves per millimeter. After preliminary pre-experimental optimization, we determined that 10 s is the optimal spectral acquisition duration, as this yields better spectral quality. For each sample, a moderate collection method was used to ensure that no significant sample evaporation, solute crystallization, or thermal denaturation occurred within 10 s under the applied laser power. This prevents signal fluctuations caused by excessive detection, thereby ensuring the stability and reproducibility of the SERS signal. Spectral collection was performed at 50 randomly selected sites for each urine sample. The spectral range was 400 cm^-1^ to 1600 cm^-1^.

### Data analysis

Label-free SERS spectral data were obtained from urine samples of LN and NS patients. LabSpec software was used for baseline correction to improve data accuracy and comparability. Further processing was done in the R programming language. All spectral data were normalized using peak area.

To construct a classification model capable of distinguishing between urine samples from different diseases, we employed supervised machine learning algorithms. During the modeling process, we used the leave-one-out method for model validation; each time, one sample was used as the test set, while the remaining samples formed the training set; this process was iterated until all samples had been predicted once. A random forest model was built based on the training set and used to predict the left-out test sample. The model performance was evaluated by plotting the receiver operating characteristic (ROC) curve, which illustrates the model discriminative ability at different thresholds by charting the relationship between the true positive rate and false positive rate. We also used box plots, violin plots, and clustering heat maps to show the intensity values of characteristic peaks. To clarify the biological significance of the characteristic peaks, we referred to previous studies on urinary Raman spectroscopy and assigned substances to each characteristic peak position. The results are shown in **Table 3**.

**Table 3:** Assignments of SERS peaks for urine metabolites.

SERS peak position (cm^-1^)	Metabolite
536, 756	Tryptophan (16, 17)
721	Hypoxanthine (18)
785	Cytosine (19)
813	Glutathione (20)
1048	Creatinine (21)
1080	Nitrogenous compounds (21)
1106	L-Leucine (22)
1131	Uric acid (23)
1204	Nucleic acids (24)
1275	Amide III (25)
1355	Isoleucine (26)
1561	N-methyladenosine (19)

## Results

### Characterization of AgNPs

**Figure 1:**
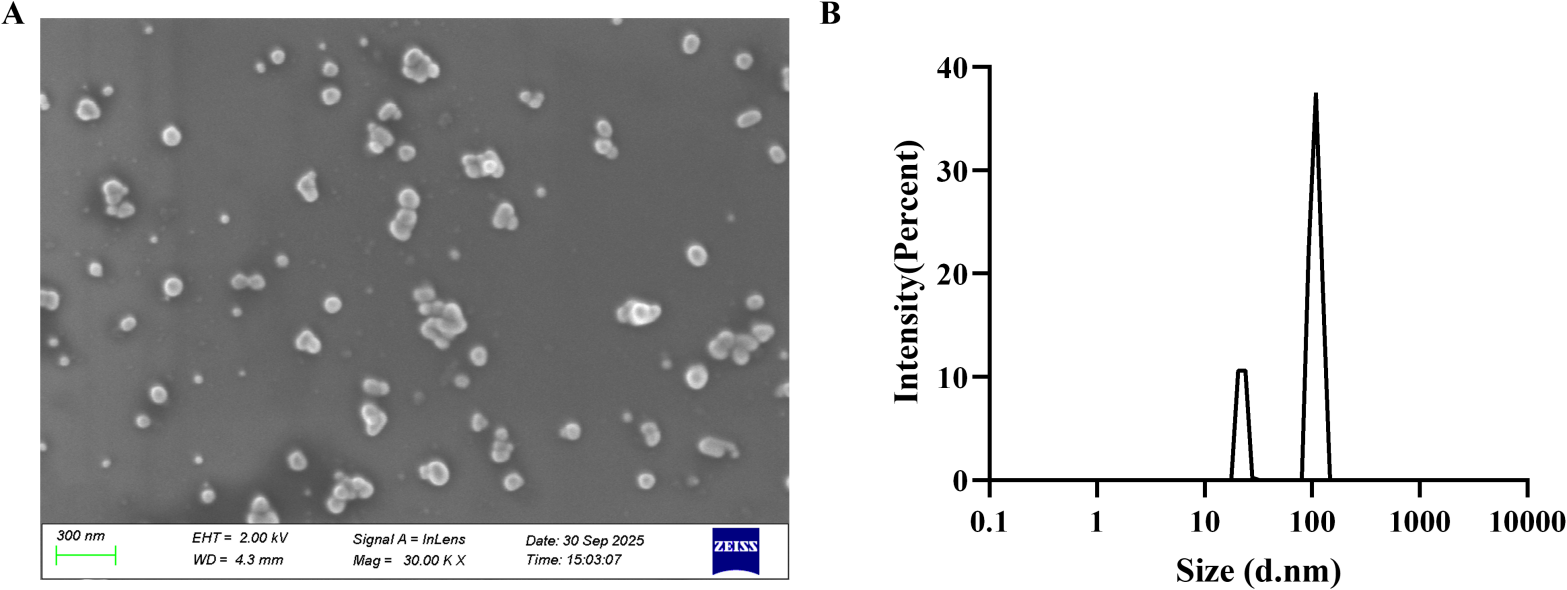
Characterization of AgNPs. **(A)** SEM of AgNPs. The scale bar is 300 nm. **(B)** Size distribution of AgNPs.

**Figure 1A** shows a scanning electron microscope image of AgNPs on a silicon wafer, where the particles look spherical or rod-shaped. **Figure 1B** presents the dynamic light scattering intensity distribution. The intensity signal exhibits a bimodal distribution, with the main peak at 107.9 nm (78.51%) and the secondary peak at 22.28 nm (21.49%).

### SERS spectral analysis of urine samples

**Figure 2:**
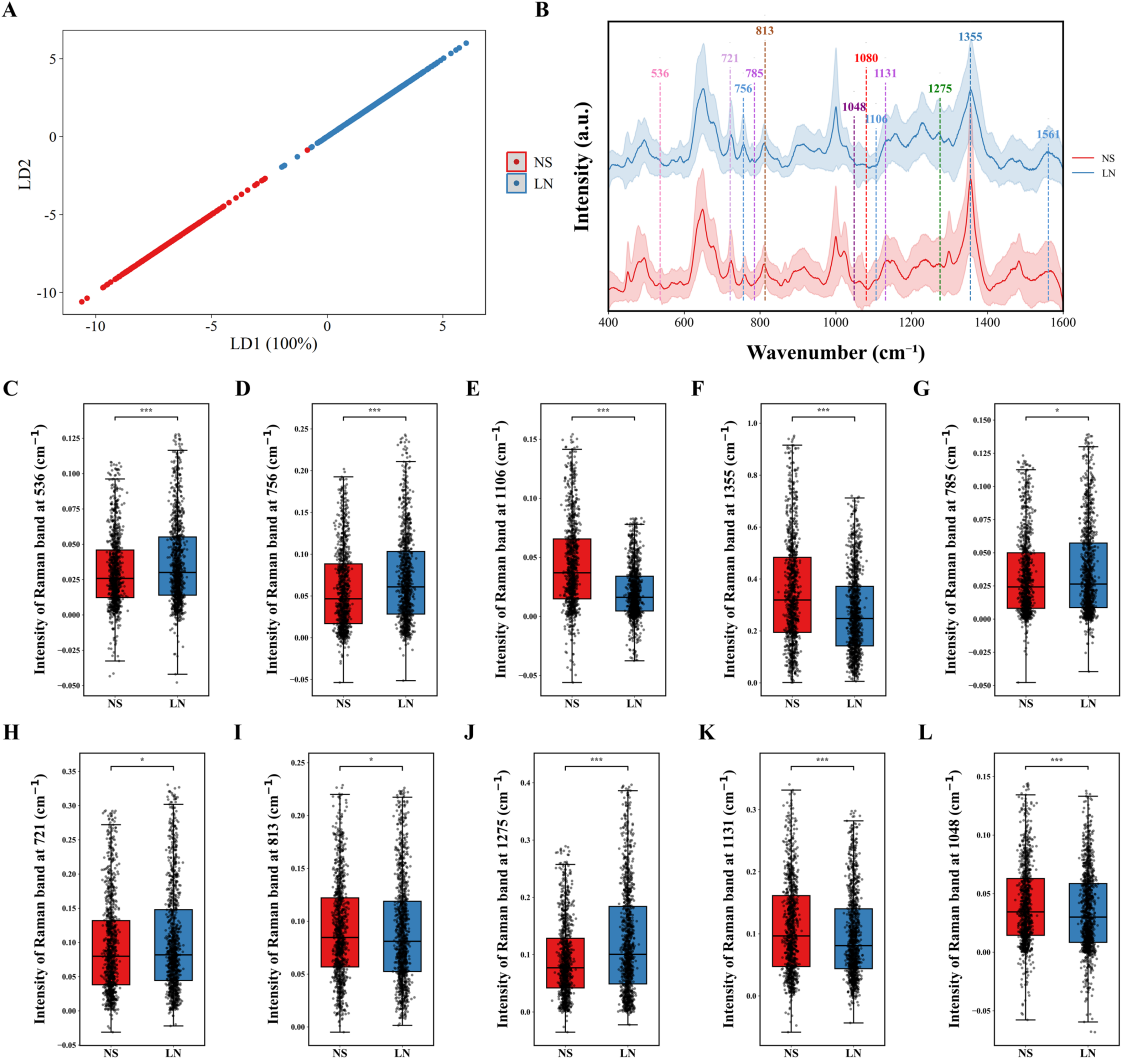
NS and LN surface-enhanced Raman spectroscopy **(A)** LDA analysis. **(B)** Average SERS with labeled differential characteristic peaks. **(C -L)** Box plot of components in urine samples, NS (red) and LN (blue). * P<0.05, *** P<0.001.

We performed systematic SERS analysis of urine samples from patients with NS and LN, focusing on the molecular characteristics and heterogeneity of urine from patients with LN. A simple and efficient test method was employed: a urine sample, without any pretreatment, was directly dropped onto an optimized SERS substrate for detection. To clarify the molecular composition differences between the NS and LN groups, this study first collected the average Raman spectra of the two groups in the 400–1600 cm^-1^ fingerprint region. To verify the ability of spectral features to distinguish between groups, a linear discriminant analysis (LDA) model was constructed based on the complete spectral data. As shown in **Figure 2A**, this axis completely separates the NS (red dots) from the LN (blue dots) groups, with no overlap between the two groups, confirming that SERS features can effectively and reliably distinguish the two sample groups.

Based on this, the study marked characteristic SERS peaks with significant intergroup differences on the average spectrum (**Figure 2B**), including 536, 721, 756, 785, 813, 1048, 1106, 1131, 1275, and 1355cm^-1^. These peaks correspond to the vibrational modes of core biomolecules, such as proteins, lipids, nucleic acids, and carbohydrates, providing a molecular basis for analyzing the molecular differences between the groups. **Figures 2C, D** are box plots of characteristic peaks at 536 cm^-^¹ and 756 cm^-^¹, respectively. The results show significant differences in tryptophan content in the urine of patients with LN compared to patients with NS (P<0.001). The characteristic peaks were located at 1106 and 1355 cm^-1^, corresponding to the characteristic peaks of leucine and isoleucine, respectively, and exhibited completely opposite expression trends in the two diseases (**Figures 2E, F**, P<0.001). This phenomenon suggests that, although both diseases may lead to significant proteinuria, there are distinct disease-specific differences in the amino acid composition of urine. For the characteristic peaks at 785 cm^-^¹ and 721 cm^-1^ (**Figures 2G, H**, P<0.05), although both correspond to important bases that make up nucleic acids (DNA and RNA), there are significant differences in these two peaks in the urine of patients with LN and NS. The typical peak at 813 cm^-^¹ may correspond to glutathione, indicating that the levels of three amino acids in the urine of patients with LN -glutamic acid, cysteine, and glycine-may differ significantly from those in patients with NS (**Figure 2I**, P<0.05). The amide III peak at 1275 cm^-^¹ is a characteristic vibrational peak for proteins and peptides in SERS, and the signal intensity of this peak in the urine of LN patients also shows significant differences compared to those of NS patients (**Figure 2J**, P<0.001). At the characteristic peaks of 1131 cm^-1^ and 1048 cm^-1^, the relative abundances of uric acid and creatinine exhibited notable variations among patients diagnosed with distinct disease types (**Figures 2K, L**, P<0.001). In conclusion, through the analysis of specific characteristic peaks in the patient’s urine sample, we can gain insight into the patient’s metabolic process.

### Machine learning distinguishes different diseases

**Figure 3:**
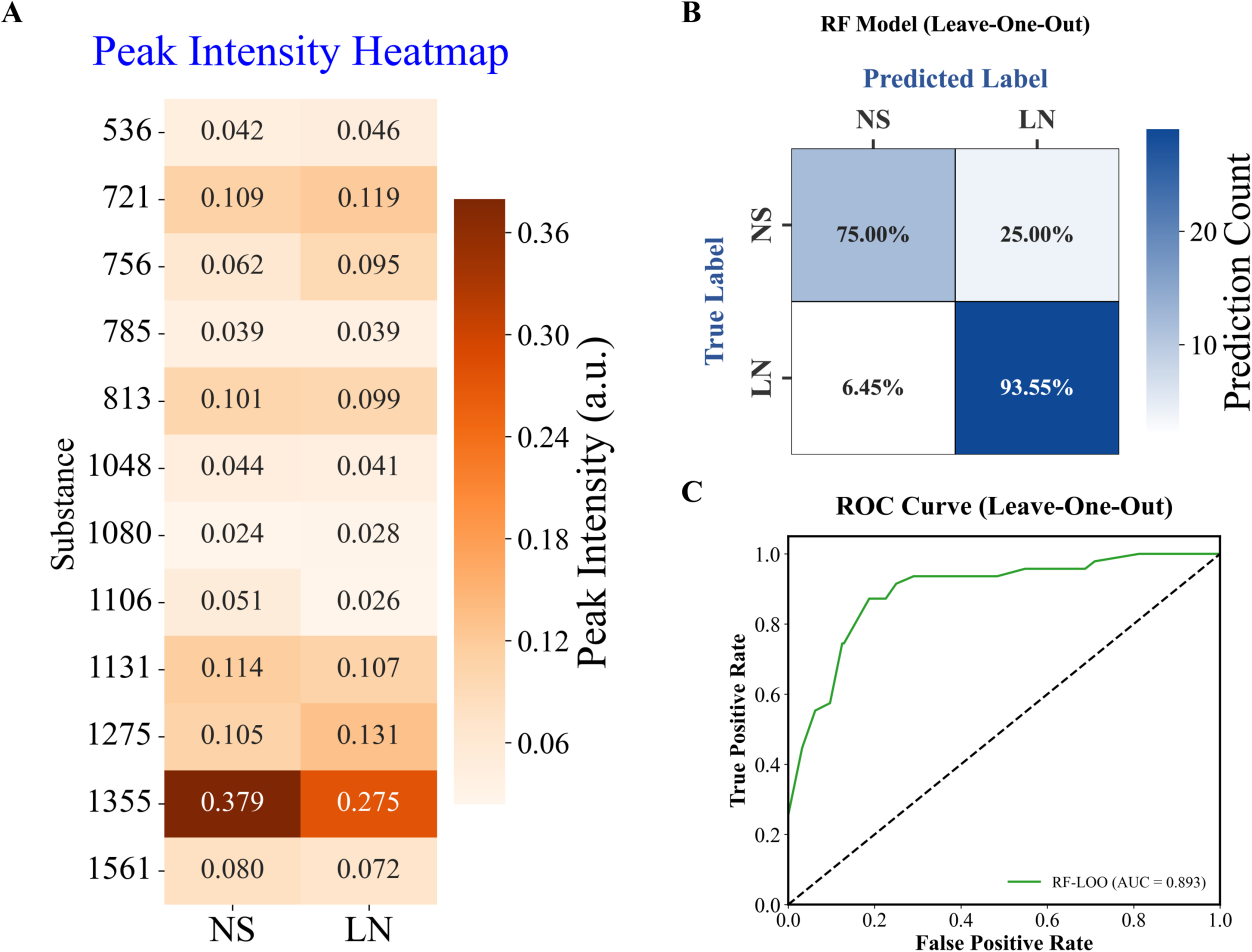
Machine Learning of NS and LN **(A)** Heatmap of 12 variables. **(B)** Confusion matrix of Random Forest Model. **(C)** AUC values.

To better understand the relationship between changes in proteins in urine samples and different diseases, we used grouping clustering heatmaps. The heat map shows the average results of the SERS peak site intensity values for the 12 urinary metabolites (**Figure 3A**). The different colors in the heat map indicate the peak site intensity values of the metabolites. The different rows and columns represent the patient’s disease type and metabolites, respectively. The distribution of various metabolites in urine samples from patients with different diseases can be observed. A significant increase in components potentially associated with proteins was observed in the urine of patients with LN, with tryptophan and glutathione being the most abundant.

Combined with SERS detection technology and a Random Forest Model, this study successfully established a classification model that can distinguish urine samples from patients with different diseases. **Figure 3B** shows the confusion matrix of the test set, showing that the urine sample accuracy is 93.55% in LN patients and 75.00% in patients with NS. These data reflect the high performance of our model on the test dataset. The ROC curve showed a high AUC value, demonstrating that the model performed well in differentiating between urine samples from different diseases (**Figure 3C**).

### SERS curves for different pathological types of lupus nephritis

**Figure 4:**
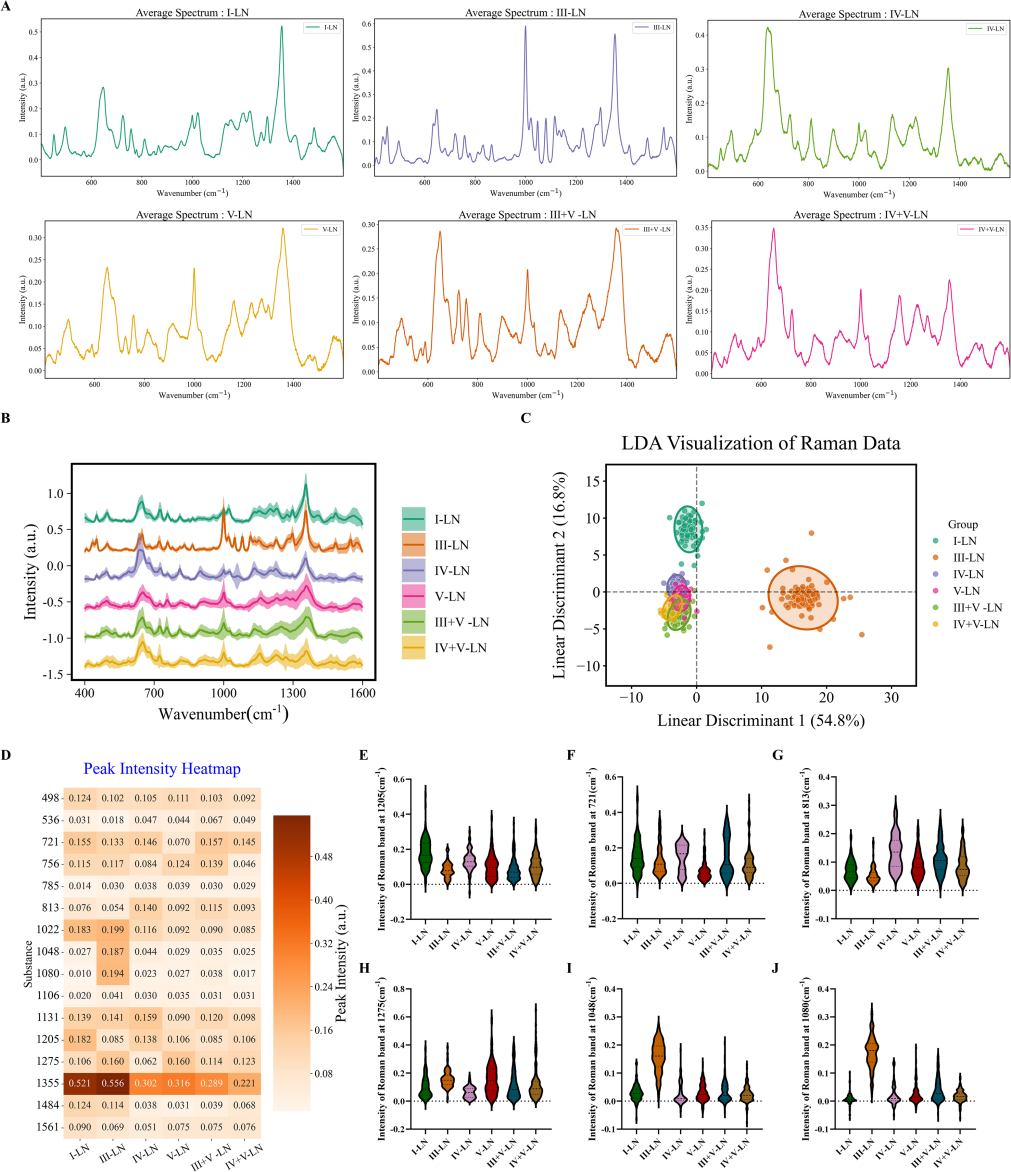
SERS curves for different types of renal pathology **(A)** Mean SERS of different renal pathology type. **(B)** Summary of different SERS. **(C)** LDA analysis. **(D)** Heatmap of 16 variables. **(E-J)** Violin plots of components in urine samples.

We classified patients according to their renal pathology results, categorizing them as follows: Minimal mesangial lupus nephritis (I-LN), Focal lupus nephritis (III-LN), Diffuse lupus nephritis (IV-LN), Membranous lupus nephritis (V), and composite III+V-LN, IV+V-LN. The intra-group differences between the six groups of samples in the mean plot are very obvious, and it can be seen that the peak position of III-LN is significantly different from that of the other samples at approximately 1100 cm^-1^ (**Figures 4A, B**). Similar results are evident in the LDA plot, where classes I and III are clearly distinct from the other species (**Figure 4C**). We further performed principal component analysis (PCA) for the six pathological groups. However, PCA showed limited separation among the six subgroups (**Figure S1**)

We performed cluster analysis of urine test results from patients with six different types of kidney pathologies (**Figure 4D**). At the 1205 cm^-1^ characteristic peak related to nucleic acids, the spectral features of type I-LN were significantly different from those of the other pathological types (**Figure 4E**, P<0.001). At the 721 cm^-1^ characteristic peak related to hypoxanthine, the peak signal of type V-LN was significantly different compared with all other experimental groups (**Figure 4F**, P<0.001). In contrast, at the 813 cm^-1^ characteristic peak related to glutathione, the relative peak intensity of type IV-LN was significantly higher than that of all other cohorts, with the difference reaching a highly significant statistical level (**Figure 4G**, P<0.001). At the 1275 cm^-1^ characteristic peak related to amide III, the relative peak intensity of IV-LN was significantly lower than that of all other experimental groups (**Figure 4H**, P<0.001). Notably, type III-LN samples exhibited specific characteristic peaks at 1048 cm^-1^ and 1080 cm^-1^, which are speculated to originate from creatinine or nitrogen-containing compounds (amines, mainly ethanolamine) (21), further peak intensity analysis showed that the relative intensity of this characteristic peak in the III-LN group was significantly higher than that of other groups, with the difference being highly statistically significant (**Figures 4I, J**, P<0.001).

## Discussion

This study analyzed urine samples from patients with lupus nephritis and nephrotic syndrome using surface-enhanced Raman spectroscopy, revealing significant differences in urine composition between the two diseases. The random forest algorithm, a powerful ensemble learning-based machine learning method, constructs multiple decision trees and employs a voting mechanism, allowing it to effectively process high-dimensional spectral data and avoid overfitting. We input the normalized spectral features of the collected urine samples into the random forest model and successfully built a highly accurate and robust diagnostic model. This model not only achieved excellent classification performance but also identified key Raman feature peaks through feature importance analysis, providing a scientific basis that combines predictive power and biological interpretability for the early and precise diagnosis of lupus nephritis.

Tryptophan metabolism is positively correlated with disease activity in SLE patients (27). Previous experiments have shown that the ratio of picolinic acid to tryptophan can serve as a new potential marker for LN diagnosis and pathological classification (28). Notably, SERS showed a significant increase in the intensity of tryptophan characteristic peaks in the urine of patients with lupus nephritis, which is highly consistent with previous studies. Ethanolamine is a key participant in phospholipid metabolism and a major component of cell membranes (29). According to previous studies, the specific peaks observed at 1048 cm^-1^ and 1080 cm^-1^ in the urine of patients with type III-LN are mostly characteristic vibrational peaks of ethanolamine. This result suggests that patients with type III-LN exhibit specific disorders in renal phospholipid metabolism or amino acid metabolism, and ethanolamine may serve as a potential biomarker for distinguishing type III-LN from other renal pathological types. The underlying mechanism may be associated with cell membrane damage induced by focal glomerular inflammation.

The kidneys are the primary tissues involved in the uptake of glutathione from the blood (30). Immunoglobulin A nephropathy is often accompanied by a decrease in glutathione (31), and genetic variations in glutathione metabolism-related enzymes may promote oxidative stress in glomerulonephritis (32). Therefore, we speculate that it may also be possible to determine the type of nephritis by detecting the peak strength of glutathione in the urine of patients with lupus nephritis. The research results show that the characteristic peaks of glutathione in the urine of patients with type IV-LN differ significantly from those of other pathological types, which may provide evidence for distinguishing between different pathological classifications.

SLE features abnormal production of autoantibodies that target nucleic acids and their binding proteins. Anti-double-stranded DNA antibodies and anti-Sm antibodies, which target RNA-binding proteins, are typical examples. These are often accompanied by various other autoantibodies (33). Nuclease inactivation or mutation can promote autoimmune diseases, suggesting that nucleic acid-related inhibitors may be potential therapeutic agents for such diseases (34). SERS detected a significant increase in the characteristic peak intensity of nucleic acids in the urine of patients with type I-LN. Therefore, it is important to further explore whether interventions targeting nucleic acids will affect the prognosis of patients with this subtype of LN.

## Conclusions

This study established a novel non-invasive liquid biopsy strategy for LN by combining label-free SERS with machine learning technology, using urine as the detection sample. Urine SERS spectral features in the 400–1600 cm^-^¹ fingerprint region showed significant differences between patients with LN and those with NS, with multiple characteristic peak intensities exhibiting statistically significant differences. Using a random forest model and leave-one-out cross-validation, this method achieved a diagnostic accuracy of 93.55% for LN and 75.00% for NS, with an AUC of 0.893, demonstrating good discriminatory power between the two types of proteinuric kidney diseases. The study identified several key diagnostic characteristic peaks for LN, each corresponding to different urinary metabolites. In addition, specific spectral markers for LN pathological subtypes were successfully identified: type I-LN showed an abnormal signal at 1205 cm^-1^, type III-LN exhibited specific increases at the 1048 and 1080 cm^-1^ peaks, type IV-LN displayed characteristic changes at 813 cm^-1^ and 1275 cm^-1^, and type V-LN showed significant differences at 721 cm^-1^, indicating the potential for effective stratification of the major pathological subtypes of LN. In summary, the SERS-machine learning platform enables non-invasive diagnosis and pathological subtyping of LN through urinary metabolic fingerprints, potentially providing a novel technological approach for clinical non-invasive detection, dynamic monitoring, and personalized diagnosis and treatment of LN.

Although the above results provide preliminary evidence for the potential application of this method in the differential diagnosis of lupus nephritis, this study remains exploratory. Its clinical practicality still needs to be verified through large-scale, prospective, multicenter cohort studies. In the future, we will combine SERS technology with mass spectrometry analysis to conduct metabolomics studies on the same batch of samples, in order to validate the identities of key discriminative molecules. This will allow for a more comprehensive assessment of the application prospects of this technology in renal pathology monitoring, provide a basis for the continuous optimization of the prediction model, and ultimately offer more precise and personalized diagnosis and treatment services for patients with lupus nephritis.

## Data Availability

The original contributions presented in the study are included in the article/**Supplementary Material**. Further inquiries can be directed to the corresponding authors.
