# Supplementary material for: Surface-enhanced Raman spectroscopy-based liquid biopsy for diagnosis and classification of lupus nephritis using urine biomarkers
Source: Front Immunol. 2026 May 5;17:1808890. doi: 10.3389/fimmu.2026.1808890 (PMC13183620; doi:10.3389/fimmu.2026.1808890)
Supplement: Supplementary file 1 [file SupplementaryFile1.docx]

**Supplementary Materials**

**
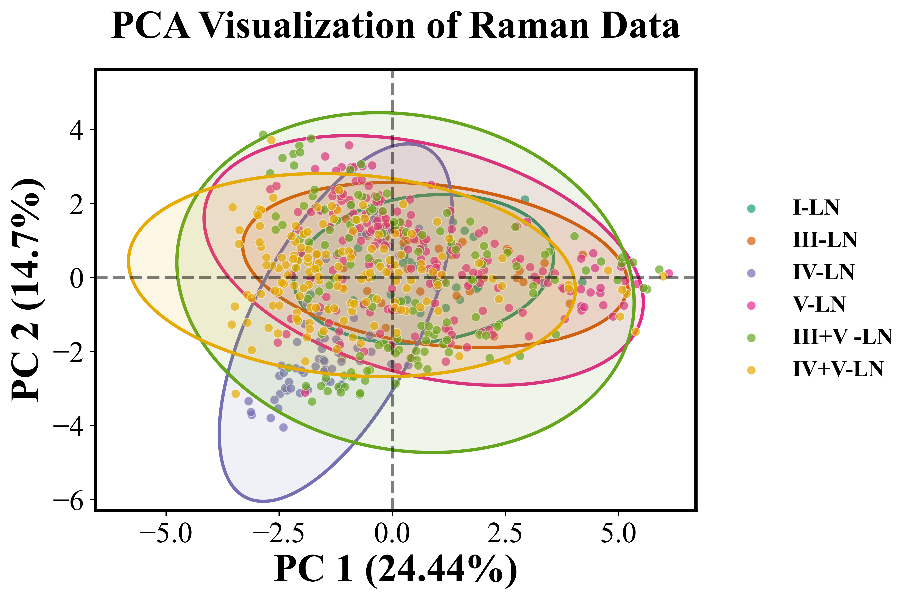
**

**Figure S1. Exploratory PCA Analysis of LN Subtypes**

Patients with different pathological subtypes of LN were included in the analysis, with distinct colors assigned to each subtype. Due to the limited sample size, this analysis is exploratory, and reliable comparisons across subtypes require validation in larger patient cohorts.
